# O.1.2-4 Evaluation of a multifaceted implementation plan for a computer-tailored physical activity intervention: a mixed methods study

**DOI:** 10.1093/eurpub/ckad133.091

**Published:** 2023-09-11

**Authors:** Denise Peels, Janet Boekhout, Femke Van Nassau, Lilian Lechner, Catherine Bolman, Brenda Berendsen

**Affiliations:** Open Universiteit, The Netherlands; Open Universiteit, The Netherlands; Amsterdam UMC, The Netherlands; Open Universiteit, The Netherlands; Open Universiteit, The Netherlands; Maastricht University, The Netherlands; denise.peels@ou.nl

## Abstract

**Background:**

Although there are many proven effective physical activity (PA) interventions for older adults, implementation in a real world setting is often lacking, limiting their actual impact on public health. This study describes the systematic development of a multifaceted implementation plan and evaluates its use during implementation of the evidence-based computer-tailored Active Plus PA intervention. Important lessons learned during this process will be presented.

**Methods:**

The implementation plan was developed following the steps of Implementation Mapping, linking multilevel determinants of implementation to implementation theory and strategies. Municipalities (n = 33) were invited to implement the intervention among their inhabitants aged 65 years and older. Quantitative and qualitative data were gathered within municipalities to assess the implementation process, i.e. achievement of the performance objectives (i.e. sub-behaviours of implementers) and utilization of implementation strategies. We also assessed implementation determinants (i.e. perceptions of the intervention characteristics, the inner setting of the organization and the outer setting) before, during and after utilization of the implementation plan.

**Results:**

A total of 8 municipalities (i.e. 24% adoption) implemented Active Plus, and 624 people aged over 65 participated in the intervention (8% adoption). Implementation strategies were used adequately. Execution of the performance objectives for adoption and implementation was relatively high (75-100%). Maintenance objectives were executed to a lesser degree (13-63%). No positive changes in implementation determinants were observed and none of the municipalities decided to further continue intervention implementation.

**Conclusion:**

Implementation Mapping was found to be a useful protocol to develop and evaluate the implementation plan. Yet, implementers’ determinants did not change favorably, and municipalities did not continue intervention implementation, although implementation strategies were used as intended. We might have not addressed the most relevant determinants with the selected implementation strategies. Whereas the current implementation plan mainly targeted implementation determinants related to the individual and the perceived intervention characteristics, a stronger focus on strategies targeting implementation determinants related to the inner setting, the outer setting and the implementation process itself is recommendable. In addition, it is advisable to use a more iterative approach, evaluating goals and determinants regularly, and consequently adapting the required implementation strategies.

